# Distribution of physical activity facilities in Scotland by small area measures of deprivation and urbanicity

**DOI:** 10.1186/1479-5868-7-76

**Published:** 2010-10-18

**Authors:** Karen E Lamb, Neil S Ferguson, Yang Wang, David Ogilvie, Anne Ellaway

**Affiliations:** 1 MRC Social and Public Health Sciences Unit, 4 Lilybank Gardens, Glasgow, G12 8RZ, UK; 2 Department of Civil Engineering, University of Strathclyde, John Anderson Building, 107 Rottenrow, Glasgow, G4 0NG, UK; 3 MRC Epidemiology Unit and UKCRC Centre for Diet and Activity Research (CEDAR), Box 296, Institute of Public Health, Forvie Site, Robinson Way, Cambridge, CB2 0SR, UK

## Abstract

**Background:**

The aim of this study was to examine the distribution of physical activity facilities by area-level deprivation in Scotland, adjusting for differences in urbanicity, and exploring differences between and within the four largest Scottish cities.

**Methods:**

We obtained a list of all recreational physical activity facilities in Scotland. These were mapped and assigned to datazones. Poisson and negative binomial regression models were used to investigate associations between the number of physical activity facilities relative to population size and quintile of area-level deprivation.

**Results:**

The results showed that prior to adjustment for urbanicity, the density of all facilities lessened with increasing deprivation from quintiles 2 to 5. After adjustment for urbanicity and local authority, the effect of deprivation remained significant but the pattern altered, with datazones in quintile 3 having the highest estimated mean density of facilities. Within-city associations were identified between the number of physical activity facilities and area-level deprivation in Aberdeen and Dundee, but not in Edinburgh or Glasgow.

**Conclusions:**

In conclusion, area-level deprivation appears to have a significant association with the density of physical activity facilities and although overall no clear pattern was observed, affluent areas had fewer publicly owned facilities than more deprived areas but a greater number of privately owned facilities.

## Background

Obesity rates are rising in industrialised countries, nearly a quarter of adults in the UK are obese[1] with higher rates observed among low income groups (particularly women)[2]. As obesity prevalence has increased, there is some evidence that there has been a simultaneous decline in levels of physical activity[3], and efforts to increase physical activity levels by changing individuals' behaviour have had limited success[4]. It has been suggested that taking an ecological approach to the obesity epidemic may be more useful[2] and that 'Understanding, measuring, and altering the "obesogenic" environment is critical to success'[5]. Obesogenic environments are those which promote excessive food intake and discourage physical activity. UK policy documents acknowledge the potential of the local physical environment to influence obesity and physical activity levels and advocate strategies aimed at 'creating supportive environments' such as access to opportunities for physical activity[6-8].

Although some studies have found obesity[9,10] and physical activity[11,12] to be associated with where people live, the precise mechanisms through which the physical environment influences obesity and physical activity are not well understood[13]. Since obesity and associated health outcomes are more prevalent among lower income groups, it is important to further our understanding of the contribution of the local environment to creating and maintaining inequalities in obesity and obesity-related behaviours[2]. One potential contributory factor is the extent to which facilities for physical activity are equitably distributed between neighbourhoods. Lack of access to facilities may have greater impact upon the health of people in deprived areas, who may face both financial and mobility barriers to using private facilities or facilities further afield[14]. Knowledge of the extent to which access and use of physical activity facilities and their associations with obesity are socially patterned is therefore important for informing the direction and focus of public health and planning policies.

A few studies (mostly conducted outside the UK) have explored the distribution of objective features of local environments such as parks or recreational facilities. Some have found fewer resources in deprived areas, while others have not[13]. In Australia, for example, lower socioeconomic status (SES) areas in Perth had better access to sports and recreation centres, gyms and swimming pools, while higher SES areas had better access to golf courses and beaches[15]; in Melbourne, on the other hand, the number and total area of free-access, restricted-access and sporting or recreational open spaces did not vary by neighbourhood SES[16]. Similarly, the GLOBE study in the Netherlands found no significant association between proximity to sports facilities and neighbourhood SES[17]. In Glasgow, Scotland there were more publicly owned swimming pools and sports centres in deprived areas, whereas publicly owned tennis and bowling clubs and private swimming pools were more prevalent in more affluent areas[18].

National studies on this topic are rare. A national study in the USA found that higher SES areas were better served with physical fitness facilities, membership-based sports and recreation clubs, dance facilities and public golf courses; these facilities were less likely to be present in areas with higher proportions of African American, Hispanic or other ethnic minority backgrounds[19]. In New Zealand, on the other hand, sports facilities were found to be more easily accessed from the most deprived neighbourhoods[20]. The only such study in the UK to date found that the availability of facilities such as gyms, swimming pools and sports halls declined with increasing area-level deprivation across England[21].

In this paper, we address the following questions: to what extent does the distribution of physical activity facilities vary by area-level deprivation in Scotland; does this patterning persist after adjusting for urbanicity (to take into account proximity to population centres potentially containing a variety of facilities); and how is the distribution of facilities patterned between and within the four largest Scottish cities? We think it is important to examine these issues as it has been suggested that, even *after *taking individual socio-economic circumstances into account, Scots may have higher rates of poor health than their English counterparts[22,23] and that between-city differences in health and health behaviours have been found for Scottish cities and regions[24,25].

## Methods

A list of all recreational physical activity facilities in Scotland and their 8-digit British National Grid references (precise to 100 metres)[26] was obtained from **sport**scotland, the national agency for sport in Scotland[27]. **sport**scotland draw this information from a variety of sources, including lottery funding applications, local authority data and publications, press cuttings; internet searches and websites. The database thus contains a snapshot of information on facilities across Scotland at a point in time (in this case June 2007). The list of facilities included 63 different classifications including both permanent facilities (e.g. football pitches, tennis courts, bowling greens, golf courses) and other facilities used intermittently for physical activity (e.g. school and church halls designated as 'occasional sports halls'). The name and address and co-ordinates of each facility and category of ownership (public i.e. local authority owned, or private club) was also recorded in the data set. The grid references of a small number of facilities were found to be incorrect. These facilities were therefore geo-located using postal addresses. As the data set includes all facilities across Scotland it was not possible to validate the data. However, in an attempt to adjust for any potential discrepancies in the **sport**scotland database, duplicate facility types (e.g. multiple squash courts within the one sports centre) were omitted prior to carrying out the analysis.

### Spatial scale

The data were mapped using Geographic Information System (GIS) software and linked to information on the datazone in which each physical activity facility was located. Datazones (DZs), formed from groups of output areas for the 2001 Census, are the key small-area statistical geography in Scotland[28]. They are nested within local government boundaries, and where possible they have been defined in such a way as to respect physical boundaries and natural communities and contain households with similar social characteristics.

There are 6,505 DZs in Scotland, with a mean population of 778 (range 476-2813). For each DZ, the publicly available 2006 Scottish Index of Multiple Deprivation (SIMD) Current Income sub-domain scores[29], determined by the proportion of individuals within an area who are income deprived, and the Scottish Executive six-fold Urban Rural Classification[30] were obtained. The SIMD is a continuous measure of compound social and material deprivation, calculated using employment, welfare benefits, health, education, housing and similar data for each DZ. The full index was not adopted in our analysis as it includes information on geographical access to services, which might have introduced a degree of circularity to an investigation of determinants of access to physical activity facilities. The SIMD scores for Scotland were grouped into quintiles (Q1 = most affluent, Q5 = most deprived). The Urban Rural Classification ranges from large urban areas (category 1) to remote rural areas (category 6), as described in Table 1, includes proximity to nearest small town.

**Table 1:** Urban Rural classification descriptions.

Urban Rural classification	Description	Population size
1	Large urban areas	Over 125,000 residents

2	Other urban areas	10,000-125,000 residents

3	Accessible small towns	3,000-10,000 residents within 30 minute drive of a settlement of an urban area

4	Remote small towns	3,000-10,000 residents with more than 30 minute drive to urban area

5	Accessible rural areas	<3,000 residents within 30 minute drive of an urban area

6	Remote rural areas	<3,000 residents with more than 30 minute drive to urban area

The physical activity facilities were grouped into public, private and 'other ownership' categories, as well as 'individual' and 'group' facility types. The public physical activity facilities consisted of local authority, community enterprise, voluntary body and trust sites; the private facilities consisted of private, club, commercial and hotel facilities; those categorised as 'other ownership' consisted of those found within schools and churches which can only be accessed for physical activity at certain times, university and college facilities which can predominantly only be accessed by those with an affiliation to these establishments, and facilities found within workplaces. The 'individual' facilities consisted of those deemed capable of being used by individuals to exercise alone and include swimming pools, weights rooms and athletics tracks. The 'group' facilities consisted of those deemed likely to be used by two or more individuals together and include football pitches, tennis courts and hockey pitches. The 'individual' and 'group' categories were created as we wished to distinguish opportunities for physical activity which people could participate by themselves (e.g. swimming) from those undertaken with others (e.g. tennis, football) as there may be different target groups of individuals for these types of facilities.

Population data from the 2001 Census small area estimates for each DZ were obtained from the General Register Office for Scotland[31]. The DZs in which no facilities were present were also included in the analysis. Some sites (such as sports centres or community centres) included several facilities of the same type (e.g. 5 football pitches) at the same location. Duplicate facility types at a single site (e.g. multiple squash courts within the one sports centre) were removed from such sites prior to analysis. The number of facilities (in total and broken down by ownership and individual/group categories) found within each Income SIMD quintile was calculated, as well as the mean density of each facility per 1,000 individuals in the DZs in each quintile.

### Statistical analysis

The statistical analysis was carried out using SPSS version 15.0. Poisson regression and negative binomial regression, which adjusts for over-dispersion in the data, were used to model the association between the number of facilities (in total and broken down by ownership and individual/group categories) and Income SIMD quintile. Negative binomial regression was adopted in the modelling of all physical activity facilities (including the separate models for the four largest city local authorities, see below) and in the modelling of the 'other ownership' facilities due to the presence of over-dispersion. Poisson regression was used in all other models. In order to adjust for differences in population size in the DZs, an offset of the natural logarithm of the population size was used in the model. This is a standard approach taken when modelling rates using count data[32]. Adjustment for population size was carried out in order to identify DZs with greater or less provision per head as poorer areas may be relatively more densely populated but may not benefit from better access to local PA facilities. To adjust for urbanicity and account for differences between the 32 local authorities, indicator variables for Urban Rural Classification and local authority were entered in the models. Multilevel modelling techniques were also explored in the analysis as DZs are nested within local authorities. However, the final results obtained for income deprivation were not altered on taking this modelling approach. Pairwise Bonferroni adjusted multiple comparisons were carried out to identify statistically significant differences in the estimated marginal mean numbers of facilities in the deprivation quintiles. The trend is not reported as the assumption of a linear association between the ordinal explanatory variable income deprivation and the response variable was not found to be valid.

## Results

### Distribution of physical activity facilities by income deprivation quintile

In total, 10,283 physical activity facilities were included in the analysis, consisting of 3,280 (31.9%) public facilities, 2,234 (21.7%) private facilities and 4,769 (46.4%) 'other ownership' facilities (see Table 2, column 1). Amongst the public and private facilities, 1,245 (22.6%) were classed as 'individual' facilities and 3,769 (68.4%) as 'group' facilities. 500 (9.1%) of the public and private facilities were not included in either group as they could not easily be classified as an 'individual' or 'group' facility.

**Table 2:** Distribution of PA facilities by Income SIMD quintile.

		Column 1	Column 2	Column 3
		**Number**	**Mean number per 1,000 residents (95% C.I.)**	**Mean number per 1,000 residents adjusting for urbanicity and local authority (95% C.I.)**

***All PA facilities***				

**Income SIMD quintile**	1 (most affluent)	1942	1.87 (1.74, 2.01)	1.99 (1.82, 2.15)

	2	2581	2.61 (2.43, 2.78)	2.55 (2.35, 2.74)

	3 (middling)	2478	2.48 (2.31, 2.65)	2.63 (2.43, 2.83)

	4	1927	1.91 (1.77, 2.04)	2.32 (2.14, 2.51)

	5 (most deprived)	1355	1.31 (1.21, 1.41)	1.87 (1.69, 2.05)

				

	Total	10283	1.98 (1.92, 2.04)	2.25 (2.14, 2.36)

			***p *< 0.001**	***p *< 0.001**

***Public facilities***				

**Income SIMD quintile**	1 (most affluent)	450	0.44 (0.40, 0.49)	0.50 (0.45, 0.55)

	2	770	0.77 (0.71, 0.82)	0.78 (0.72, 0.84)

	3 (middling)	859	0.86 (0.80, 0.91)	0.91 (0.84, 0.97)

	4	684	0.68 (0.63, 0.73)	0.81 (0.74, 0.88)

	5 (most deprived)	517	0.50 (0.46, 0.54)	0.71 (0.63, 0.78)

				

	Total	3280	0.63 (0.61, 0.65)	0.73 (0.69, 0.76)

			***p *< 0.001**	***p *< 0.001**

***Private facilities***				

**Income SIMD quintile**	1 (most affluent)	501	0.49 (0.45, 0.54)	0.55 (0.49, 0.61)

	2	730	0.73 (0.68, 0.78)	0.65 (0.59, 0.71)

	3 (middling)	543	0.54 (0.50, 0.59)	0.55 (0.49, 0.60)

	4	301	0.30 (0.26, 0.33)	0.39 (0.34, 0.44)

	5 (most deprived)	159	0.15 (0.13, 0.18)	0.23 (0.19, 0.26)

				

	Total	2234	0.39 (0.37, 0.41)	0.44 (0.41, 0.47)

			***p *< 0.001**	***p *< 0.001**

***Other ownership facilities***				

**Income SIMD quintile**	1 (most affluent)	991	0.94 (0.86,1.02)	0.88 (0.79, 0.97)

	2	1081	1.09 (1.00, 1.18)	0.99 (0.89, 1.08)

	3 (middling)	1076	1.07 (0.98, 1.16)	1.05 (0.95, 1.15)

	4	942	0.93 (0.85, 1.01)	1.03 (0.92, 1.13)

	5 (most deprived)	679	0.66 (0.60, 0.72)	0.84 (0.74, 0.94)

				

	Total	4769	0.92 (0.89, 0.96)	0.95 (0.89, 1.01)

			***p *< 0.001**	***p *= 0.003**

***Individual facilities***				

**Income SIMD quintile**	1 (most affluent)	245	0.24 (0.21, 0.27)	0.30 (0.26, 0.35)

	2	331	0.33 (0.30, 0.37)	0.32 (0.28, 0.36)

	3 (middling)	328	0.33 (0.29, 0.36)	0.35 (0.30, 0.39)

	4	202	0.20 (0.17, 0.23)	0.28 (0.24, 0.32)

	5 (most deprived)	139	0.14 (0.11, 0.16)	0.23 (0.19, 0.28)

				

	Total	1245	0.23 (0.22, 0.25)	0.29 (0.27, 0.32)

			***p *< 0.001**	***p *= 0.005**

***Group facilities***				

**Income SIMD quintile**	1 (most affluent)	644	0.64 (0.59, 0.68)	0.71 (0.64, 0.77)

	2	1072	1.07 (1.01, 1.13)	1.04 (0.96, 1.11)

	3 (middling)	945	0.94 (0.88, 1.00)	0.99 (0.92, 1.07)

	4	671	0.66 (0.61, 0.71)	0.81 (0.75, 0.88)

	5 (most deprived)	437	0.42 (0.38, 0.46)	0.59 (0.52, 0.65)

				

	Total	3769	0.71 (0.69, 0.73)	0.81 (0.77, 0.85)

*P*-values presented indicate whether or not a statistically significant difference is present between at least one pair of deprivation quintiles.

In univariate analysis, quintile 2 (Q2) had the highest number of physical activity facilities, with 25% of all facilities located in DZs with this classification (see Table 2, column 2). In addition, Q2 had the highest number of private (33% of the total), 'other ownership' (23%), 'individual' (27%) and 'group' (28%) facilities of all Income SIMD quintiles. The highest number of public facilities (26% of the total) was found in the middling Income SIMD quintile (Q3).

Following adjustment for population, Urban Rural classification and local authority, Q3 had the highest estimated density of facilities at 2.63 per 1,000 residents (see Table 2, column 3). Bonferroni adjusted pairwise multiple comparisons of the estimated marginal means were carried out. No statistically significant difference was found in the mean number of all facilities between the most affluent (Q1) and most deprived (Q5) quintiles. However, these two groups had statistically significantly lower estimated mean numbers of all facilities than all other quintiles.

The more affluent quintile (Q1) had a significantly lower estimated mean number of public facilities than all other quintiles. Considering the private physical activity facilities, both Q4 and Q5 had lower estimated mean number of private facilities (after adjustment) compared to the other quintiles, with Q5 showing the lowest. Q5 also had the lowest estimated mean number of 'other ownership' facilities (significantly different from Q3 and Q4).

The only statistically significant differences in the estimated mean number of 'individual' facilities were between Q2 and Q5 and between Q3 and Q5.

### Distribution of physical activity facilities by city

The local authorities representing the four largest cities in Scotland (Glasgow, Edinburgh, Dundee and Aberdeen) were examined separately to investigate differences in the number, density and distribution of physical activity facilities between and within the cities (Table 3). After adjusting for population size, Dundee and Edinburgh had the highest estimated density of facilities whilst Glasgow had the lowest. Only Aberdeen and Dundee had statistically significant within-city differences by deprivation. The mean number of PA facilities decreases with increasing deprivation from Q2 to Q5 for Dundee, with the only statistically significant differences observed between Q1 and Q5 and Q2 and Q5. Both Q1 and Q2 have significantly higher means than Q5. This pattern was not observed in Aberdeen where the mean number of facilities is highest in Q3, with statistically significant differences found between Q1 and Q3 and between Q5 and Q3.

**Table 3:** Density of PA facilities for Aberdeen, Dundee, Edinburgh and Glasgow.

Local authority		Number of DZs	Number of PA facilities	Mean number of facilities per 1,000 residents (95% C.I.)
***Aberdeen***				

**Income SIMD quintile**	1 (most affluent)	90	108	1.48 (1.07, 1.90)

	2	43	68	1.91 (1.18, 2.64)

	3 (middling)	45	127	3.56 (2.34, 4.77)

	4	57	85	1.83 (1.22, 2.45)

	5 (most deprived)	32	32	1.31 (0.66, 1.95)

				

	Total	267	420	1.89 (1.57, 2.21)

				***p *= 0.001**

***Dundee***				

**Income SIMD quintile**	1 (most affluent)	30	63	2.53 (1.43, 3.64)

	2	23	71	3.55 (1.87, 5.24)

	3 (middling)	15	32	3.18 (1.22, 5.13)

	4	35	39	1.37 (0.75, 2.00)

	5 (most deprived)	76	63	1.04 (0.69, 1.38)

				

	Total	179	268	2.10 (1.66, 2.54)

				***p *< 0.001**

				

***Edinburgh***				

**Income SIMD quintile**	1 (most affluent)	190	321	1.94 (1.59, 2.29)

	2	122	222	2.29 (1.78, 2.79)

	3 (middling)	83	150	2.23 (1.63, 2.82)

	4	73	111	1.93 (1.36, 2.50)

	5 (most deprived)	81	122	1.80 (1.29, 2.30)

				

	Total	549	926	2.03 (1.80, 2.26)

				***p *= 0.623**

				

*Glasgow*				

**Income SIMD quintile**	1 (most affluent)	29	43	1.60 (0.84, 2.35)

	2	82	78	1.16 (0.80, 1.53)

	3 (middling)	84	69	0.98 (0.67, 1.30)

	4	128	114	1.09 (0.82, 1.37)

	5 (most deprived)	371	362	1.18 (1.01, 1.35)

				

	Total	694	666	1.19 (1.02, 1.36)

				***p *= 0.553**

*P*-values presented indicate whether or not a statistically significant difference is present between at least one pair of deprivation quintiles.

Considering a model of the facilities for the four city local authorities alone, adjusting for population and deprivation, Glasgow had a statistically significantly lower number of facilities overall than the other three cities. On further investigation of the public, private, 'other ownership', 'individual' and 'group' breakdown (data not shown), it appears that the difference is predominantly in the 'other ownership' facilities such as facilities within work places, schools, community centres, town halls and churches.

## Discussion and conclusions

Although a statistically significant association between area-level income deprivation and the number of physical activity facilities was identified, there was no clear pattern. This is generally consistent with the mixed picture obtained from other studies [13,15,17,18,33]. It does appear that the most affluent and most deprived areas have fewest facilities overall. However, there is a difference when examining this by facility ownership with the more affluent areas appearing to have the lowest density of public facilities but amongst the highest density of private facilities.

One possible interpretation of our findings is that the sociospatial patterning of PA, obesity and health related outcomes may be more strongly associated with the nature, quality and affordability of PA facilities rather than with simple quantitative measures of the presence of facilities. Another is that sociospatial gradients in other factors not represented in the **sport**scotland dataset, such as opportunity, motivation and self-efficacy to make use of PA facilities, are more important than any sociospatial gradient in access to facilities as such. Other studies have found that the mere provision of opportunities for physical activity is insufficient to encourage their use [34].

In comparison to our study, the study of the association between physical activity facilities and deprivation in England showed a clearer pattern with the availability of facilities declining with increasing deprivation after adjustment for population size[21]. However, there were a number of differences between this study and our Scottish study that make it difficult to draw direct comparisons. In particular, we included outdoor as well as indoor facilities, used different area deprivation indices and spatial units, and adjusted for degree of urbanicity.

The strength of this study is that the data are representative of a wide range of physical activity facilities present in Scotland, and demonstrates how a routine data set collected by public bodies for other purposes can be used for a statistically sophisticated piece of public health analysis. There are, however, some limitations to the study. We assessed the availability of facilities in Scotland, but there was no information available on the quality of the facilities listed in the data set; there may have been differences due to the variety of methods used by **sport**scotland to collect the data, although we made adjustments to the dataset by removing duplicate facilities of the same type within sites prior to analysis; the dataset represents the facilities available at a particular point in time and may be incomplete; and the **sport**scotland database only covers 'sports' facilities and does not include other facilities or locations where individuals may undertake recreational (or other) physical activity such as parks, mountains, beaches, traffic-free cycle routes, etc.

Furthermore, in the analysis of all physical activity facilities no distinction was made between those exclusively used for physical activity, such as tennis courts or bowling greens, and those only used for occasional physical activity purposes, such as occasional small sports halls within community centres. However, by examining the public, private and 'other ownership' facilities separately a picture can be obtained of the facilities available for regular use in Scotland as all community centres and church halls were grouped in the 'other ownership' category. Finally, the statistical relationships we found may have been altered if different area levels had been selected for the analysis, an issue known as the modifiable areal unit problem (MAUP)[35]. To avoid this problem, and to take into account the fact that the opportunity to use PA facilities does not end at DZ boundaries, the analysis could have been improved by including road networks to permit an analysis of the accessibility of facilities by different modes of transport such as walking, cycling, public transport and car. This would reflect the fact that those with suitable means of transportation, such as car access or good quality public transport, particularly in more urban contexts, are likely to have a greater number and variety of PA facilities accessible to them. We are currently undertaking this more detailed analysis and will report it in a future paper.

## List of abbreviations

C.I.: confidence interval; UK: United Kingdom; USA: United States of America.

## Competing interests

The authors declare that they have no competing interests.

## Authors' contributions

AE, DO and NSF conceptualised the study, KEL performed the statistical analysis, NSF and YW mapped the PA facilities and linked them to datazones. All authors contributed to the drafting of the manuscript and approved the final version.
